# Supplementary figures and images for: Mycobacterial heparin-binding hemagglutinin (HBHA)-induced interferon-γ release assay (IGRA) for discrimination of latent and active tuberculosis: A systematic review and meta-analysis
Source: PLoS One. 2021 Jul 16;16(7):e0254571. doi: 10.1371/journal.pone.0254571 (PMC8284824; doi:10.1371/journal.pone.0254571)

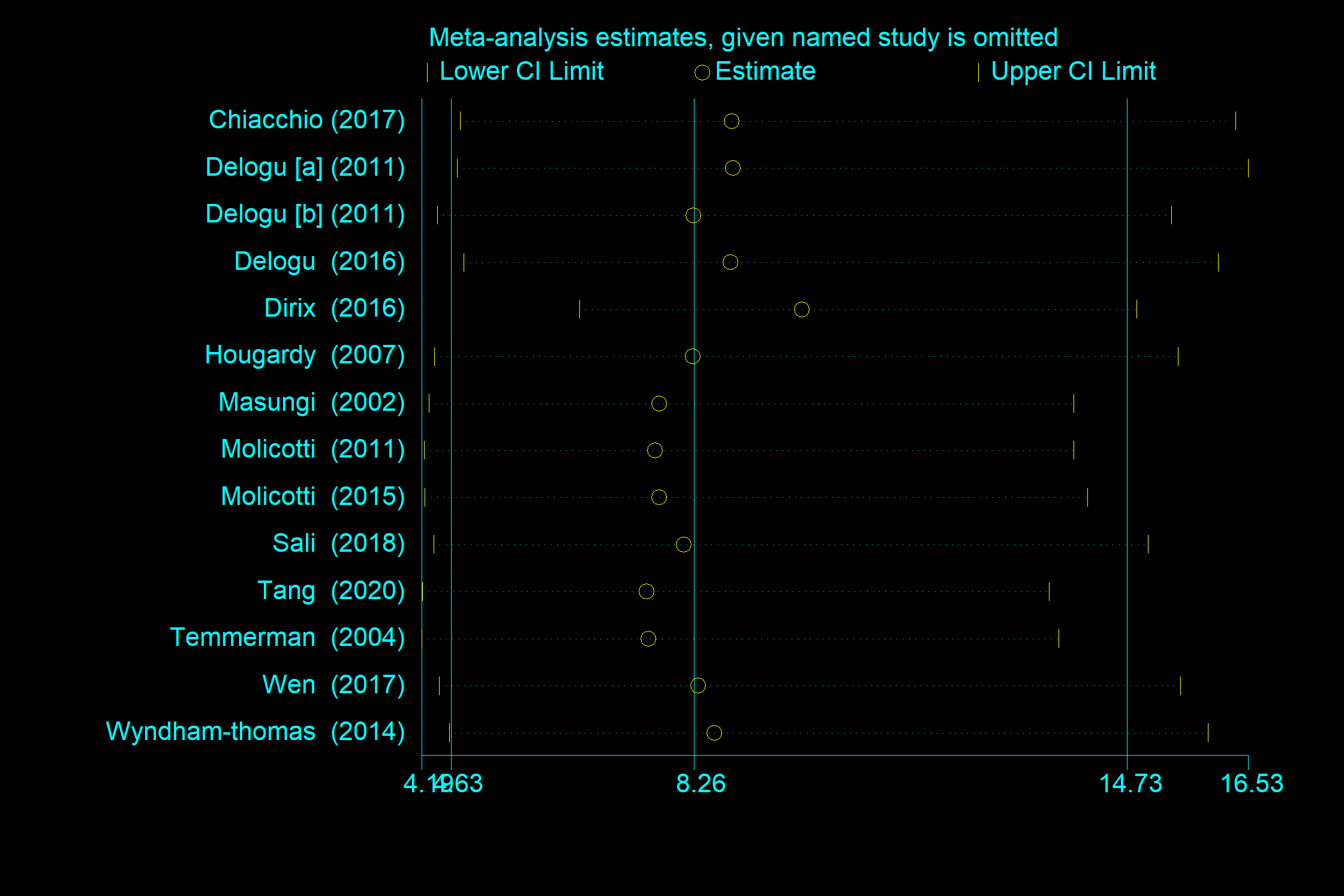

Supplement: S1 Fig — (TIF) [file pone.0254571.s002.tif]

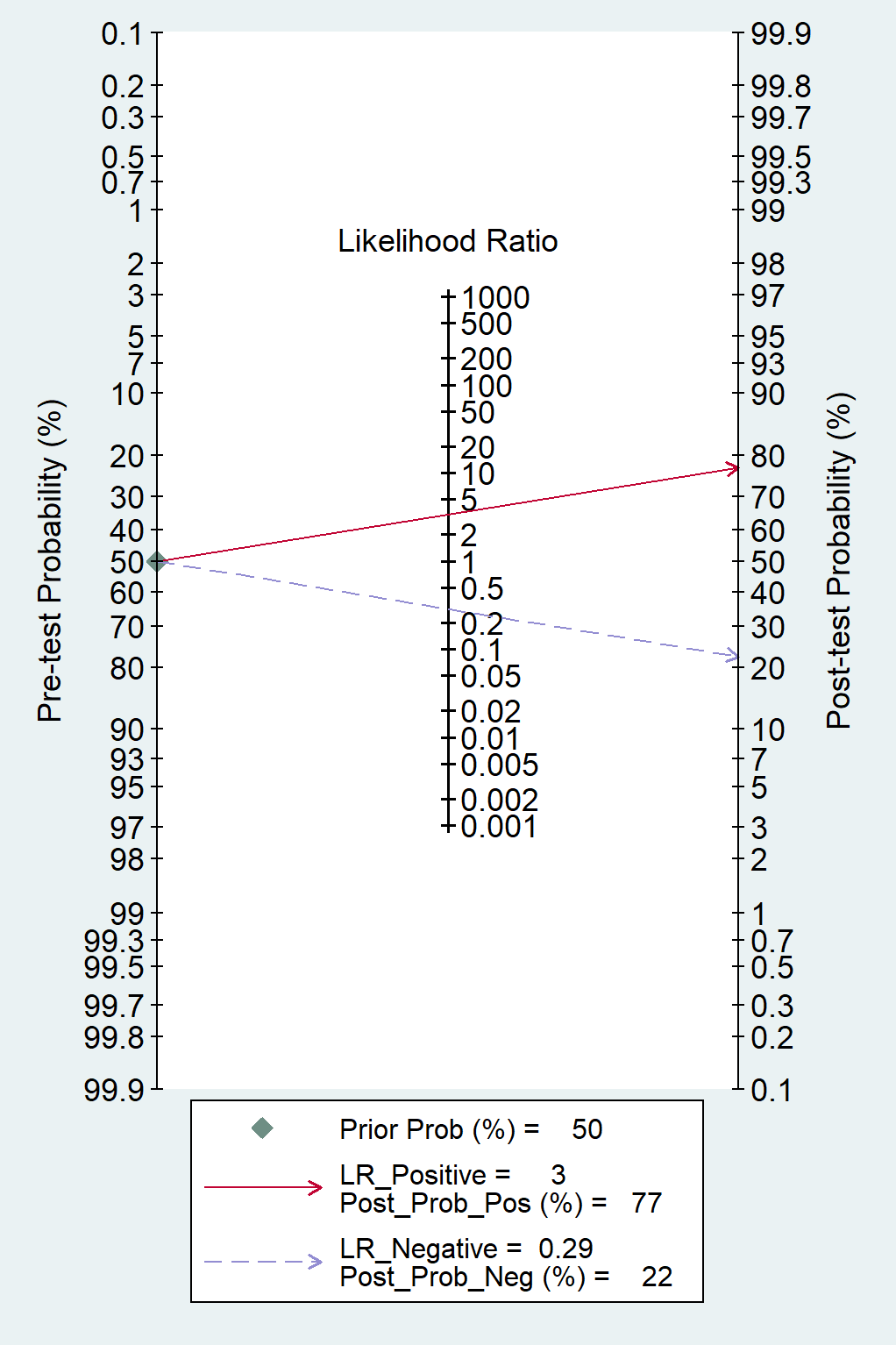

Supplement: S2 Fig — Pre-test probability = 50%. (TIF) [file pone.0254571.s003.tif]
