# Supplementary material for: Mycobacterial heparin-binding hemagglutinin (HBHA)-induced interferon-γ release assay (IGRA) for discrimination of latent and active tuberculosis: A systematic review and meta-analysis
Source: PLoS One. 2021 Jul 16;16(7):e0254571. doi: 10.1371/journal.pone.0254571 (PMC8284824; doi:10.1371/journal.pone.0254571)
Supplement: Attachment — Submitted filename: Response to Reviewers.docx [file pone.0254571.s005.docx]

**Response to the reviewers*'* comments**

Dear Dr. Olivier Neyrolles and Reviewers:

Thank you for your and the reviewers’ comments concerning our manuscript entitled “Mycobacterial heparin-binding hemagglutinin (HBHA)-induced interferon-γ release assay (IGRA) for discrimination of latent and active tuberculosis: a systematic review and meta-analysis” (Manuscript Number: PONE-D-21-06709R1). Your comments and those of the reviewers were highly insightful and enabled us to greatly improve the quality of our manuscript. We have studied comments carefully and have made corrections which we hope meet with approval. The revised portion are marked in the Revised Manuscript with Track Changes. The responds to the reviewer’s comments are as follows:

**Reviewer #1:** (No Comments)

**Reviewer #2:**

**Comments for the author**

please, in table 1, in the column indicating TYPE OF TB, regarding the papers by:

1. Delogu, Plos One 2011

2; Chiacchio, PloS One, 2017:

please indicate that the type of TB was PULMONRY TB in 100% of the TB patients evaluated, as stated in the material and method sections where it is written that TB was microbiologically diagnosed on sputum.

Moreover, in the paper by Sali et al, you may see in Table 1, that extrapumonary TB is in 5 over 19 (26%)

**Response:** Thank you very much for your reminder of the omissions in our paper. The related revised portions are marked in the paper.

**Reviewer #3:** The authors appropriately answer to my comments and I only have a few remaining comments.

**Comments for the author**

1. Abstract:

a. Results – line 40: why did you replace “did” by “may”? I think “did” was more appropriate

**Response:** Thank you for your advice. The related revised portion is marked in the paper.

b. Conclusion. I suggest to delete the last sentence that was added. It is unnecessary and does not correspond to the main message of your meta-analysis. You clearly show that the HBHA-IGRA is very robust and minimally influenced by various technical differences between the studies. Therefore, it is not appropriate to conclude that large and high quality studies are further needed. What is necessary now as mentioned at the end of your discussion, is to arise the interest of a commercial company.

**Response:** Thanks. Your comment is right. The last sentence here may cause misunderstanding and confusion. Therefore, we delete this sentence according to your suggestion.

2. Discussion:

a. I still have a concern concerning the interpretation of the results obtained in studies included in your meta-analysis and comprising HIV-infected patients. You conclude that low responses in these patients may be due to the low CD4+ T cell number in these patients (lines 259-262). However, Wyndham-Thomas et al who were in 2015 the first to evaluate the performance of the HBHA-IGRA in HIV-infected people living in a low TB incidence country, reported that the HBHA-IGRA was more sensitive that both the TST and QFT test to identify potentially Mtb infected people (3 subjects with an isolated HBHA-IGRA had a high Mtb exposure risk). In addition, they showed that the 3 HIV-infected patients with a positive HBHA-IFN-g response had very high responses contrasting to what is reported for non-HIV infected subjects. This observation does not sustain the hypothesis that low CD4+ T cell number account for the low sensitivity of the HBHA IGRA often reported in HIV-infected subjects.

**Response:** Considering your suggestion, we have substantially elucidated both the outcome of subgroup analysis in HIV-infected people and the observation in the study of Wyndham-Thomas et al in 2015 in the discussion. we consider that the reasons for the low sensitivity of the HBHA-IGRA in HIV-infected patients are complex, and suggest that the mechanism of HIV infection affecting HBHA-induced IFN-γ release still needs further investigation. The specific mechanism is not the main focus of the discussion in this paper.

b. Lines 290-291: variability of the cut-off between different studies may also be due to different demographic characteristics of the populations included in the studies.

**Response:** We support your suggestion. As you suggested, the related discussion has been added in the limitation.

3. Conclusion

a. Lines 323-324…I suggest “…combination of the results of the HBHA-IGRA with those from other IGRAs (ESAT-6 and CFP-10-based) may allow optimal stratification of *Mtb* infected patients in different groups with variable risks of reactivation of the infection. Currently, the HBHA-IGRA is the only….”

**Response:** Thank you for your suggestion. The related revised portion is marked in the paper.

b. line 330-331. I do not understand your statement “the procedure of this test needs to be further standardized and optimized” as you clearly showed that technical differences had no impact on the diagnostic performance.

I suggest to modify this sentence as follows: “To commercialize the HBHA-based IGRA to efficiently distinguish LTBI from ATB, it is urgent to rise the interest of commercial companies to this test in order to provide kits with well defined technical conditions and cut off”

**Response:** Thank you for your suggestion. We have re-written this part according to your suggestion. The related revised portion is marked in the paper.

4. Table 3

Please correct the subgroups within active TB. I guess the 2nd group is “Clinically confirmed TB”

In the footnote of the table, Mycobacterium smegmatis should be written in italic.

**Response:** We are sorry for our incorrect writing. The related revised portions are marked in the paper.

I previously suggest the authors to cite a paper published by Smits K in 2015 and the authors did not find the reference. There was indeed a mistake and I apologize for this. The exact ref is J. Clin. Cell. Immunol. 2015; 6:4 at http://dx.doi.org/10.4172/2155-9899. 1000341

**Response:** Thank you very much for providing the specific information of the reference, the reference has been cited in this paper.

Finally, I strongly suggest the authors to have a final re-lecture and correction by a native English speaking people as the English language should really be improved mostly, but not exclusively, for the modified sentences in the revised version of the manuscript.

**Response:** Thanks for pointing out our language problem. We employed an English-language editing service, MedSci, to polish our wording. Certification is attached.

Kind regards,

First Author: Jinhua Tang

Corresponding author: Yueyun Ma

E-mail addresses: [mayueyun2020@163.com](mailto:mayueyun2020@163.com)

Clinical Laboratory

Air Force Medical Centre

30 Fucheng Road, Beijing, China 100142
